# Predicting falls in older adults: an umbrella review of instruments assessing gait, balance, and functional mobility

**DOI:** 10.1186/s12877-022-03271-5

**Published:** 2022-07-25

**Authors:** D. Beck Jepsen, K. Robinson, G. Ogliari, M. Montero-Odasso, N. Kamkar, J. Ryg, E. Freiberger, Masud Tahir

**Affiliations:** 1 Geriatric Research Unit, Department of Clinical Research, University of Southern Denmark, Odense, Denmark; 2 Department of Geriatric Medicine, Odense University Hospital, Odense, Denmark; 3 Department of Health Care for Older People (HCOP), Research and Innovation, Queen’s Medical Centre, Nottingham University Hospitals NHS Trust, Nottingham, UK; 4 School of Medicine, University of Nottingham, Nottingham, UK; 5 Gait and Brain Lab, Parkwood Institute, SLawson Health Research Institute, London, ON Canada; 6 Division of Geriatric Medicine, Department of Medicine, Schulich School of Medicine & Dentistry, University of Western Ontario London, London, ON Canada; 7 Department of Epidemiology and Biostatistics, Schulich School of Medicine & Dentistry, University of Western Ontario, London, ON Canada; 8 Institute for Biomedicine of Aging, FAU Erlangen-Nürnberg, Nuremberg, Germany

**Keywords:** Accidental Falls, Gait; Balance, Function, Older Adults, Fall Prediction, Umbrella review

## Abstract

**Background:**

To review the validated instruments that assess gait, balance, and functional mobility to predict falls in older adults across different settings.

**Methods:**

Umbrella review of narrative- and systematic reviews with or without meta-analyses of all study types. Reviews that focused on older adults in any settings and included validated instruments assessing gait, balance, and functional mobility were included. Medical and allied health professional databases (MEDLINE, PsychINFO, Embase, and Cochrane) were searched from inception to April 2022. Two reviewers undertook title, abstract, and full text screening independently. Review quality was assessed through the Risk of Bias Assessment Tool for Systematic Reviews (ROBIS). Data extraction was completed in duplicate using a standardised spreadsheet and a narrative synthesis presented for each assessment tool.

**Results:**

Among 2736 articles initially identified, 31 reviews were included; 11 were meta-analyses. Reviews were primarily of low quality, thus at high risk of potential bias. The most frequently reported assessments were: Timed Up and Go, Berg Balance Scale, gait speed, dual task assessments, single leg stance, functional Reach Test, tandem gait and stance and the chair stand test. Findings on the predictive ability of these tests were inconsistent across the reviews.

**Conclusions:**

In conclusion, we found that no single gait, balance or functional mobility assessment in isolation can be used to predict fall risk in older adults with high certainty. Moderate evidence suggests gait speed can be useful in predicting falls and might be included as part of a comprehensive evaluation for older adults.

**Supplementary Information:**

The online version contains supplementary material available at 10.1186/s12877-022-03271-5.

## Background

Over one-third of adults aged 65 years and older fall at least once a year [1]. Increasing age, frailty, comorbidity, impaired gait, muscle weakness, and impaired balance all contribute to the risk of falls [2]. Falls are a major cause of disability and constitute the leading cause of injury-related mortality in people aged above 75 years [3]. The importance of an individualised approach to screening, assessment, and intervention is emphasised across professional guidelines such as the Steadi Algorithm [4]. There is no clear consensus on the specific choice of fall assessment; however, professional guidelines state that adults at high risk should be able to access individually tailored multifactorial measures based on a comprehensive assessment [5, 6]. This should include assessment of gait, balance, and motor function with targeted interventions to address any limitation since these domains are associated with an increased risk of falls [7, 8]. Assessing these limitations could help to identify older adults at risk of falling and allow targeted intervention to reduce this risk.

Multiple approaches to assess gait, balance, and functional mobility have been developed including the Berg Balance Scale (BBS), the Timed Up and Go (TUG) test, and gait speed testing, such as the dual-task gait test. Although widely used across clinical practice, there appears to be little standardisation and difficulty determining the most appropriate tool [9]. Systematic reviews of individual tools have provided limited and conflicting evidence for a tool’s predictive ability, thus precluding the ability to make clear clinical recommendations [10–13]. To this end, we performed an umbrella review to synthesize the findings across multiple systematic reviews to help develop recommendations for clinical practice.

The aim of this umbrella review was to systematically review, critically appraise, and summarize the existing reviews on the use of assessment tools of gait, balance, and functional mobility to predict falls in older adults or distinguish fallers from non-fallers. This review is part of a larger initiative on behalf of the task force on global guidelines for falls in older adults (details available at https://worldfallsguidelines.com/) [14]. This paper presents a summary of the umbrella review for Working Group 1, and the findings will be fed into a wider consensus development process to develop key recommendations in the assessment and management of falls for older adults.

## Methods

This umbrella review is reported according to the Preferred Reporting Items for Systematic Reviews and Meta-analyses (PRISMA) [15] and the protocol was previously registered on PROSPERO’s international online register of systematic-, rapid-, and umbrella reviews (PROSPERO CRD42020225101).

### Search strategy

The electronic academic databases MEDLINE, PsychINFO, Embase, and the Cochrane database for Systematic Reviews were searched from inception to November 23rd, 2020. The searches search were then updated on April 20th, 2022. To ensure a broad review of available literature, no restrictions on publication date were applied. A comprehensive search strategy was developed with the support of a research librarian using a combination of medical subject heading (MeSH) terms and key words for the concepts of older adults, gait, balance, and functional mobility assessments, and falls prediction. Only studies in English were included. The full search strategy for MEDLINE is presented in Additional file 1 at the end of this document and this strategy was adapted for each of the included databases. The reference lists of included papers were also reviewed to identify any further relevant reviews for inclusion.

### Selection criteria

#### Types of studies

We included the following types of review studies:

- Narrative reviews, defined as reviews that may or may not present a systematic synthesis of findings from all individual studies included [16];
- Systematic reviews without meta-analysis, defined as having an explicit reproducible methodology including a systematic search that aims to identify all studies that meet pre-specified eligibility criteria followed by a systematic presentation and synthesis of the findings of all included studies [17];
- Systematic reviews with meta-analysis, defined as systematic reviews using statistical techniques to combine and summarize the results of multiple studies [17].

We excluded the following types of studies: conference abstracts, student theses, books, book chapters, and papers reporting empirical data from a single study rather than reviewing more than one study. Reviews which included technology-based instruments only were excluded, as there is another on-going systematic review on this topic from Working Group 8 of the task force on global guidelines for falls in older adults (PROSPERO CRD42021241177).

#### Populations and settings

We included reviews of empirical studies in older adults (women and/or men), aged 60 years or older, in any setting. Specifically, we included reviews in all the following settings: the community, and primary and secondary care settings, including long-term care institutions, rehabilitation, and acute hospital settings. We also included reviews that presented data from various age groups in case they presented data on a subgroup of older adults aged 60 years or above separately. Following this, we excluded reviews examining individuals exclusively younger than 60 years of age.

#### Assessments

Reviews that included validated assessments of gait, balance, and functional mobility to predict falls or to distinguish fallers from non-fallers.

#### Outcomes

Our primary outcome of interest was the prediction of falls. Secondary outcomes were as follows: reliability, validity including sensitivity, specificity, feasibility, and cost of the assessments.

### Study selection

Two reviewers (KR, DBJ) independently screened titles and abstracts of all records for eligibility, using the online software package Rayyan (https://www.rayyan.ai/). Disagreements were resolved by the assessment of a third reviewer (GO). Full text articles were retrieved and screened independently by two reviewers (KR, DBJ) with disagreements resolved by the assessment of a third reviewer (GO).

### Data extraction

Three reviewers (KR, DBJ, GO) extracted the data by using a pre-defined data extraction form developed specifically for this review. The following data were extracted:

- **Review details**: author(s), year of publication, country of lead author, type of participants, review objective, number of participants, age range of participants, mean age of participants, and proportion of women.
- **Search details**: sources searched, type of analysis (narrative review, systematic review without meta-analysis, or systematic review with meta-analysis), number of studies included in the review, design of studies included, and countries in which included studies were conducted.
- **Critical appraisal**: date range of included studies, critical appraisal tool(s) used in the review, and critical appraisal score.
- **Gait, balance, and functional mobility tests assessed**: fall prediction outcome, measurement of falls, predictive ability, reliability, validity (specificity, and sensitivity).
- **Cost**: any cost analysis conducted.

### Risk of bias assessment

Three reviewers (KR, DBJ, GO) assessed the risk of bias of the included studies using the Risk of Bias Assessment Tool for Systematic Reviews (ROBIS) [18]. ROBIS assesses four domains: 1) study eligibility criteria; 2) identification and selection of studies; 3) data collection and study appraisal; and 4) synthesis and findings.

### Data synthesis

To provide key clinical and research recommendations on assessment tools for fall prevention, the findings were synthesised for the most commonly reported gait, balance, and functional mobility assessments. Due to the heterogeneity of the reviews with regards to participant characteristics, settings, and assessment protocols, it was not appropriate to conduct a meta-analysis. A narrative synthesis was conducted for each gait, balance, and functional mobility assessment that was reported by more than two review studies. The narrative synthesis was conducted based on the review type and quality, as well as the number of reviews addressing this assessment and the key findings. For each review, the results were interpreted to indicate whether the findings in relation to the assessment tool’s predictive ability for falls were favourable, not favourable, inconsistent, or unclear (if data could not be extracted). An overall summary for each assessment was then made based on the highest quality available evidence. The synthesis is presented in tabular format; in the tables, the studies are ordered based on their quality.

## Results

### Search results

**Fig. 1:**
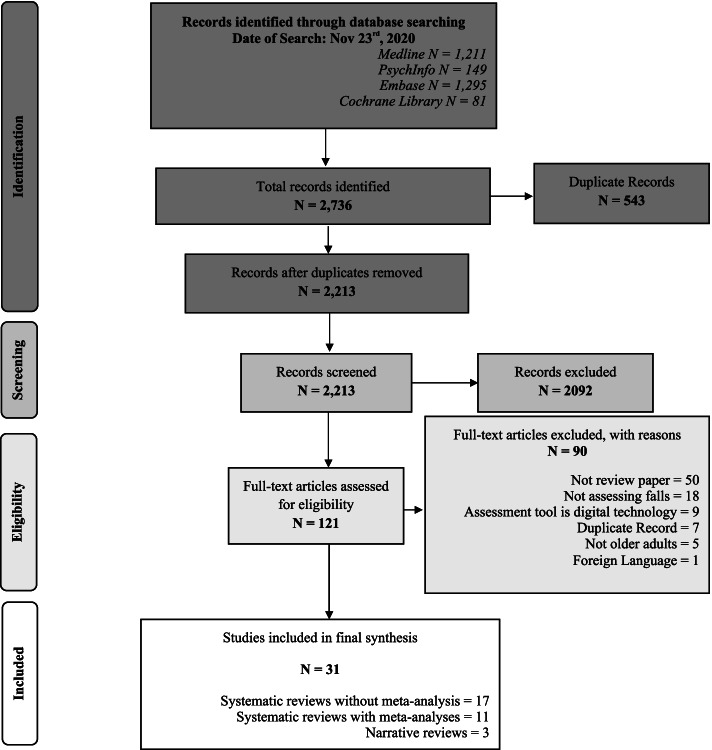
PRISMA flow chart. *Adapted From:* Moher D, Liberati A, Tetzlaff J, Altman DG, The PRISMA Group (2009). *P*referred *R*eporting *I*tems for *S*ystematic Reviews and *M*eta-*A*nalyses: The PRISMA Statement. PLoS Med 6(7): e1000097. doi:10.1371/journal.pmed1000097 For more information, visit www.prisma-statement

The literature search identified a total of 2736 potentially relevant records. Of these, 543 were duplicates. The titles and abstracts of the remaining 2213 records were screened. After excluding 2092 items in the screening, the full texts of 121 articles were assessed for eligibility. After excluding further 90 records (50 were not review papers; 18 did not assess falls; 9 were technology-based instruments only; 7 were duplicate records; 5 were not in older adults; and 1 was not in English), we included 31 records in our analyses. Figure 1 at the end of this document shows the PRISMA flow-chart.

### Characteristics of included reviews

Table 1 presents a summary of the 31 included review studies. Three were categorised as narrative reviews, 17 as systematic reviews without meta-analysis, and 11 were systematic reviews with meta-analyses. Nine reviews reported on community dwelling older adults only, one reported on long term care settings only, one reported on emergency department settings only, and 13 reported studies across a range of settings including community, supported living, residential care, outpatient and inpatient settings. Four reviews provided no details on settings. Three reviews reported that they included older adults with cognitive impairment. Healthy community-dwelling older people were the primary focus of reviews however older people with neurological disorders were included in one review [33], older people receiving inpatient stroke rehabilitation were included in one review [35], and older people being assessed in the emergency department was the focus of one review [27].

**Table 1:** Summary of included reviews

Type of review	Author (Date) Country of lead author	Reported primary objective	Study population and setting	Number of studies reporting on falls	Gait, balance and functional mobility assessment	Reported key conclusions
**Narrative Reviews**	Ambrose et al. (2015) [19] USA	To identify the epidemiology, aetiology and risk factors of fall-related fractures in older people	Not stated and no details provided on characteristics	11	Tinetti, BBS, stride length, motion centre, TUG, 5 chair stand	Clinician screening to prevent falls is recommended to identify impairments in gait and balance
	Nakamura et al. (1998) [20] USA	To review, compare and contrast the five most frequently cited scales of balance (Performance Oriented Assessment of Balance, “Get Up and Go”, Berg Balance Scale, Functional Reach Test) and Falls Efficacy (Falls Efficacy Scale) to assist clinicians in selecting an appropriate instrument for use with older adults in a clinical or research setting.	Older adults. No further details provided	Not reported	Tinetti, Get Up and Go, Functional Reach Test, the Falls Efficacy Scale, BBS	Administration time for these scales range from 3 minutes to 15–20 minutes. These scales do not require the use of expensive equipment; they require minimal space and no special training for the health care professional.
	Stasny et al. (2011) [21] USA	To assess the ability of the Activities-specific Balance Confidence (ABC) scale to predict the fall risk in older community-living adults.	Community-dwelling older adults with age range of 60–99 years	3	Activities-specific Balance Confidence Scale	Two papers showed an association between ABC scores and falls, while the third showed no associations. There is limited evidence that the ABC scale alone can predict falls.
**Systematic Reviews without meta-analysis**	Abellan Van Kan et al. (2009) [22] France	To examine if gait speed, assessed at usual pace and over a short distance, may have the capacity to identify autonomous community-dwelling older people at risk of adverse outcomes, and if gait speed might be used as a single-item tool instead of more comprehensive but time-consuming assessment instruments.	Community-dwelling older adults	4	Gait Speed	Gait speed was an independent predictor of falls or falls related femoral neck fracture in all dour studies
	Bayot et al. (2020) [23] France	To better define the role of DT in assessing the fall risk in healthy older adults, without cognitive impairment (i.e., mild cognitive impairment, dementia or neurological conditions) and/or known gait disorders.	Primarily community-dwelling healthy adults without cognitive impairment adults	30	Dual tasking	Promising added value of dual tasks including turns and other transfers, such as in the Timed Up and Go test, for prediction of falls.
	Beauchet et al. (2011) [24] France	To assess the association and the predictive ability of the TUG time performance on the occurrence of falls among individuals aged 65 years and older.	All 70 years and over Community-dwelling population (*n* = 7), inpatient population (r*n* = 3), sheltered housing (*n* = 1)	11	Timed Up and Go	Although retrospective studies found that the TUG time performance is associated with a past history of falls only one prospective study found a significant association with falls.
	Di Carlo et al. (2016) [25] Italy	Aim of this study was to provide a comprehensive review of the psychometric features of the Mini-BESTest when administered to patients with balance dysfunction	All settings, adult with balance disorders most commonly reported in studies (*n* = 19, 79%)	24	Mini-Best Test	The results support the reliability, validity, and responsiveness of this instrument and it can be considered a standard balance measure.
	Dolatabadi et al. (2018) [26] Canada	Systematic review of quantitative measures of gait and balance related to the prediction of falls, with a focus on older adults with dementia.	All settings. Older adults with diagnosis of dementia	15	TUG, 180 turn, BBS, PPT, 6 min walk, tandem gait, dynamic/static balance, POMA, mCTSIB, Romberg test, Functional Reach Test, grip strength, 4 step balance, SPPB	Limitations of gait and balance are association with increased risk of falls in cognitively intact people. The characteristics most predictive of a fall are still unclear.
	Eagles et al. (2018) [27] Canada	To identify mobility assessments that are used in ED patients of 65 years and older and determine whether mobility test measures are associated with reported outcomes of hospitalization, repeat visits to the ED, future falls, or frailty.	65 years and over undergoing mobility assessment in ED	3	TUG, Tandem Gait, Gait abnormality	No association with TUG and frailty and no falls results given despite outcome of falls reported. No association with tandem gait and future falls.
	Ganz (2007) [28] USA	To identify the prognostic value of risk factors for future falls among older patients.	Community dwelling older people	15	Anterior postural sway, self-perceived mobility, tandem stand, tandem walk, 10 m walk	The presence of at least 6 of 7 gait or balance abnormalities led to an increased risk of a fall (LR, 1.9; 95% CI, 1.4–2.6)
	Lee et al. (2013) [29] USA	To review the current evidence for fall risk screening assessments	Community-dwelling (*n* = 12) inpatient medical and surgical wards (*n* = 13), rehabilitation setting (*n* = 6)	31:	Timed Up and Go Test), Functional Gait Assessment, St Thomas Risk Assessment Tool, Hendrich fall risk model II, 10-Minute Walk Test, Berg Balance Scale, and Step Test	Timed Up and Go Test with a cut off > 12.34 seconds and Functional Gait Assessment among community-dwelling older people. St Thomas Risk Assessment Tool in medical inpatients < 65 years old and surgical inpatients; Hendrich fall risk model II in medical inpatients. 10-Minute Walk Test in patients in post-stroke rehabilitation and Berg Balance Scale or the Step Test in patients in post-stroke rehabilitation who had fallen during their inpatient stay
	Lima et al. (2018) [11] Brazil	To verify whether the BBS can predict falls risk in older adults	Community dwelling older adults (*n* = 5), older adults needing home care (reviews = 1), outpatients of geriatric clinic (*n* = 1) residential home (ns = 1).	8	BBS	The evidence to support the use of BBS to predict falls is insufficient, and should not be used alone to determine the risk of falling in older adults
	Marin-Jimenez et al. (2022) [30] Spain	To investigate the predictive validity of motor fitness and flexibility test in relation to health outcomes in adults and older adults	Healthy community-based population older than 18 years. Sub population > 65 years old	25 studies, 2 systematic reviews included falls or hip fracture	Gait speed test (13 studies+ 2 sys review), postural balance tests (13 + 1 review, adults over 40 so not included), and TUG (10 studies + 1 sys review, adults over 40 so not included)	Strong evidence for slower gait predicting falls in adults over 60 years (seven studies+ seven studies from systematic reviews) Three studies did not find an association between gait speed test and falls.
	Omana et al. (2021) [31] Canada	Systematically review the existing literature on the falls-related diagnostic test properties of the Functional Reach Test (FRT), single-leg stance test (SLST), and Tinetti Performance-Oriented Mobility Assessment (POMA) in older adults across settings and patient populations	Participants aged 60 years or more community-dwelling older adults	21 met the inclusion criteria (12 POMA, 8 FRT, 6 SLST)	Functional Reach Test (FRT), single-leg stance test (SLST), and Tinetti Performance-Oriented Mobility Assessment (POMA).	All the clinical tests of balance demonstrated an overall low diagnostic accuracy and a consistent inability to correctly identify fallers. None of these tests individually are able to predict future falls in older adults.
	Muir-Hunter et al. (2016) [32] UK	To evaluate the association between dual-task testing protocols and future fall risk	Community-dwelling participants aged 60 years and over.	10	Dual task	Changes in gait under dual-task testing are associated with future fall risk, and this association is stronger than that for single-task conditions.
	Neuls et al. (2011) [33] USA	To determine the ability of the Berg Balance Scale (BBS) to predict falls in the older people with and without pathology. Specifically, to determine the cut off score that is most predictive of falls in the older adults and the sensitivity and specificity of the BBS in predicting falls.	5 studies of healthy older adults and 4 of adults with neurological disorders	9	BBS	The Berg Balance Scale alone is not useful for predicting falls in the older adults; it should be used in conjunction with other tests or measures.
	Pamoukdjian et al. (2015) [34] France	To review the use of gait speed as a single frailty marker in older adults and to then discuss its contribution in geriatric oncology as a simple screening test for frail patients requiring a CGA (however, studies included are on community-dwelling adults, not on geriatric patients)	Living in a community setting, independent in walking	46	Gait Speed	Gait speed over a short distance is a simple, reliable, reproducible and inexpensive tool to predict falls and other adverse outcomes associated with frailty. Recommend evaluating gait speed over a distance of 4 m with a threshold of 1 m/s in a single measure as a screening tool for frailty in older patients with cancer (aged 65 years and older); those with gait speed < 1 m/s over a 4 m distance should be then assessed with a CGA.
	Scott et al. (2007) [35] Canada	To conduct a systematic review of published studies that test the validity and reliability of fall-risk assessment tools for use among older adults	Community (14 studies and 23 measures), home-support (4 studies and 4 measures), long-term care (6 studies, 10 measures), acute care (12 studies, 8 measures)	34	38 different tools were assessed in the 34 articles included in this review	There were several fall-risk assessment tools that were tested in prospective studies in different settings (community, supportive housing, long-term care, acute care). Most prospective studies assessed fall-risk assessment tools in the community setting. Yet, few tools were tested more than once or in more than one setting. Therefore, no single tool can be recommended for implementation in all settings
	Yang et al. (2015) [36] Hong Kong	To evaluate the evidence related to the psychometric properties of dual-task balance assessments in older adults.	Primarily community dwelling Mean age ranged from 69.4–81.1 years	23	Force platform	Both static and walking balance assessment tools had good reliability but were not useful to predict falls. In most of the studies, the participants were living independently and had normal cognition. The psychometric properties of dual-task assessment tools may differ depending on the cognitive status
	Zijlstra (2008) [37] The Netherlands	To evaluate whether dual-task balance assessments have an “added value” over single-task balance assessments.	Community, nursing homes, senior residences, community-centers for older adults, institutions, Alzheimer Care Units, residential care facilities and not reported in a few studies with both young and older adults	19	Dual balance tasks	Two prospective studies suggested that dual balance tasks may have added value for fall prediction over single balance tasks.
**Meta-analysis**	Barry et al. (2014) [10] Ireland	To examine the predictive value of the test to identify individuals at risk of falling	Community- dwelling older adults	25 studies (meta-analysis on 10 data sets)	TUG	Logistic regression analysis indicated that the TUG score is not a significant predictor of falls (OR = 1.01, 95% CI 1.00–1.02, *p* = 0.05)
	Beauchet et al. (2009) [24] France	To examine the relationship between the occurrence of falls and changes in gait and attention-demanding task performance whilst dual tasking amongst older adults	65 years and over; mean age ranged from 68.4–85 years. Included community –dwelling, senior housing facilities, inpatient facilities.	15	Dual task walking	Out of 3 retrospective and 8 prospective studies, two and six studies, respectively, showed a significant relationship between changes in gait performance under dual task and history of falls. The pooled odds ratio for falling was 5.3 (95%CI, 3.1–9.1) when subjects had changes in gait or attention-demanding task performance whilst dual tasking.
	Chantanachai et al. **(**2021) [38] Australia	To identify risk factors for prospectively ascertaining falls	Older people with cognitive impairment living in the community	16	TUG, gait speed, TUG-DT, pOMA, 5xSTS, static and leaning balance, limb strength, physical profile assessment, dual tasking	Mean difference in meta-analysis fallers vs non fallers TUG 2.20 (−1.42, 5.82) (4. studies) Gait speed −0.07 (−0.28,-0.06) (4 studies, (*p* = 0.46) Poor balance 0.62(0.45,0.79) (*n* = 590) *p* < 0.005 Balance impairment is a risk factor for falls in people with cognitive impairment living in the community. With less certainty, mobility and gait speed may be important risk factors for falls in this population. No met-analysis on sit-to-stand and POMA.
	Chen-Ju Fu et al. (2021) [39] Taiwan A systematic review with meta-analysis	To review whether the simple and equipment-free assessments could efficiently identify the functionally independent elderly to be fallers or non-fallers	Elderly aged over 65 years who can walk without assistance	Fifteen studies were selected for systematic review, of which nine were for meta-analysis	5-time sit-to-stand test, alternate step test, one leg stance test, functional reach test, tandem stance test, stair ascent and stair descent test, ten-step test, minimal chair height standing test, half-turn test, and maximum step length test	It was concluded that the 5-time sit-to-stand test was mostly used to assess the risk of falling in elderly. Although most assessment tests demonstrated significant difference between the fallers and non-fallers, the performance of those tests for identifying fallers were less promising.
	Kozinc et al. (2020) [40] Slovenia	Comprehensive comparison of the diagnostic balance tests used to predict falls and for distinguishing older adults with and without a history of falls	Older adults 60 years and over Mean age 74.06 ± 5.75 years. No detail on settings.	67	Single-leg stance test, body sway measures), dual body sway test and cognitive tests.	Among the non-instrumented tests, the single-leg stance test appears to be the most promising for discrimination between fallers and non-fallers. Single less stance: < 1.02 seconds – 67% sensitivity and 89% specificity
	Lusardi et al. (2017) [9] USA	Evaluate predictive ability of performance based measures for assessing fall risk by calculating and comparing PoTP values) and to explore the usefulness of PoT using results from multiple measures	Range: 65 years or over. Mean age not reported. Community-dwelling older adults.	59	56 measures. In particular, 7 performance-based measures: The Berg Balance Scale (BBS), the single-task Timed Up and Go (TUG) test, the Single-limb stance (SLS), the 5 times sit-to-stand test (5TSTS), The Performance-Oriented Mobility Assessment (POMA, Tinetti), the Self-selected walking speed (SSWS), the dynamic gait index	No single test or measure demonstrated strong PoTP values. 5 performance-based measures may have clinical usefulness in assessing risk of falling on the basis of cumulative PoTP. Berg Balance Scale score (<=50 points), Timed Up and Go times (> = 12 seconds), and 5 times sit-to-stand times (> = 12) seconds are currently the most evidence-supported functional measures to determine individual risk of future falls
	Menant et al. (2014) [41] Australia	To determine whether dual task walking paradigms involving a secondary cognitive task have greater ability to predict falls than single walking tasks	Community dwelling older adults and residents of an old age residential home, senior housing facilities or intermediate care hostels, geriatrics and Alzheimer’s care unit inpatients, geriatric out patients. 15 studies included participants with no cognitive impairment.	33 (30 in meta-analysis)	Single and dual task tests of gait speed	Findings indicate single and dual task tests of gait speed are equivalent in the prediction of falls in older people and sub-group analyses revealed similar findings for studies that included only cognitively impaired participants, slow walkers or used secondary mental-tracking or verbal fluency tasks
	Muir et al. (2010) [42] Canada	Summarize the evidence linking balance impairment as a risk factor for falls in community-dwelling older adults	Community-dwelling older adults 60 years and over	23	Tandem stand, tandem walk, one leg stand, Forward Reach Test, Performance Oriented Mobility Assessment (POMA), Berg Balance Scale, Timed Up & Go Test, Romberg Test, and body sway.	Statistically significant associations for increased falls risk identified for tandem stand, tandem walk, one leg stand, POMA and body sway.
	Park (2018) [12] South Korea	To compare the diagnostic accuracy of several currently available fall risk assessment tools developed for the older people; to identify the assessment tools most frequently used to discriminate fallers and non-fallers and the assessment tools having the highest predictive validity; to provide scientific evidence for selecting the best tool to use in practice	Older adults admitted to acute care hospitals; community-dwelling older adults; older adults in the long-term care setting. This review only included studies of people aged 60 years	33	26 tests were assessed in the 33 included studies; of these, the tests used in two or more studies were: Berg Balance Scale, Downton Fall Risk Index, Hendrich II Fall Risk Model, Mobility Interaction Fall chart, St. Thomas’s Risk Assessment Tool in Falling Inpatients (STRATIFY), Timed Up and Go test, Tinetti Balance scale.	Of the 26 tools assessed, the Berg Balance Scale has a specificity of 0.9; it is the most useful in identifying the older adults at low falls risk
	Rosa et al. (2019) [43] Brazil	To identify evidence about the usefulness of the Functional Reach Test to evaluate dynamic balance and risk of falling; to verify the FRT assessment method and other variables (anthropometric, physical) that could interfere with the test results; to establish normative values for the FRT in older adults with no specific health condition.	Community-dwelling (*n* = 31), nursing homes (*n* = 3), inpatient (*n* = 2), outpatients (*n* = 1)	40 (5 prospective studies included in meta-analysis)	Functional Reach Test	This meta-analysis provides normative values for the Functional Reach Test (FRT) (26.6 cm among community-dwelling older adults) as 15.4 cm [95%CI: 13.47;17.42] for non-community older adults (*n* = 5 studies). The meta-analysis revealed that FRT was not capable of predicting falls (*p* = 0.098). There is evidence to support the use of the FRT to assess dynamic balance but not to support its use to predict falls.
	Schoene et al. (2013) [13] Australia	To investigate the discriminative ability and diagnostic accuracy of the Timed Up and Go Test (TUG) as a clinical screening instrument for identifying older people at risk of falling	Independent community-dwelling (*n* = 40), long term care residents (*n* = 4), day care attendees (*n* = 10, outpatient clinics (*n* = 2), day hospitals (*n* = 2), geriatric inpatient (*n* = 4)	53	TUG	The Timed Up and Go Test (TUG) is not useful for discriminating fallers from non-fallers in healthy, high-functioning older people. It is of more value in less-healthy, lower-functioning older people.

### Risk of bias assessment in the included reviews

Of the 31 included reviews, ten were globally deemed at low risk of bias, eight at unclear risk of bias, and 13 at high risk of bias (Table 2). Areas of high or unclear risk of bias primarily related to limiting searches with language restrictions, selection and data extraction not done in duplicate, and a lack of quality appraisal of the individual studies.

**Table 2:** Quality assessment of the included studies according to Risk of Bias Assessment Tool for Systematic Reviews [18]

Paper	Study eligibility criteria	Identification and selection of studies	Data collection and study appraisal	Synthesis and findings	Risk of bias in the review
Abellan Van Kan [22]	Low	High	High	High	High
Ambrose [19]	Unclear	Unclear	High	High	High
Barry [10]	Low	Low	Low	Low	Low
Bayot [23]	Low	Low	Low	Low	Low
Beauchet [44]	Unclear	High	High	Low	Unclear
Beauchet [24]	Low	High	Unclear	Unclear	Unclear
Chantanachai [38]	Low	Low	Low	Low	Low
Chen-Ju Fu [39]	High	High	Low	High	High
Di Carlo [25]	Low	High	Unclear	High	High
Dolatabadi [26]	Low	Low	High	High	Unclear
Eagles [27]	Low	Low	Low	Low	Low
Ganz [28]	Low	Low	Low	Unclear	Low
Kozinc [40]	Low	Low	High	High	Unclear
Lee [29]	Low	Unclear	High	High	High
Lima [11]	Low	Low	Low	Low	Low
Lusardi [9]	Low	Low	Low	High	Unclear
Marin-Jimenez [30]	Unclear	Low	Low	Unclear	Unclear
Menant [41]	Low	Low	Low	Low	Low
Muir [42]	Low	Unclear	Unclear	Low	Low
Muir-Hunter [32]	Low	Low	Low	Low	Low
Nakamura [20]	High	High	High	High	High
Neuls [33]	High	High	High	High	High
Omana [31]	Low	Low	Low	Unclear	Low
Pamoukdjian [34]	Low	High	High	High	High
Park [12]	Low	High	Low	High	High
Rosa [43]	Low	Low	Unclear	Unclear	Unclear
Schoene [13]	High	High	Unclear	Unclear	High
Scott [35]	High	High	High	Unclear	High
Stasny [21]	High	High	Unclear	High	High
Yang [36]	High	High	Low	Low	Unclear
Zijlstra [37]	Low	Unclear	High	High	High

### Gait, balance, and functional mobility assessments

The most frequently reported gait, balance, and functional assessments for falls prediction included the following tests: TUG, BBS, tests of gait speed, dual task assessments, single leg stance, Functional Reach Test (FRT), tandem gait and the chair stand test.

#### Timed up and go

The TUG consists of a combination of standing from a chair and walking 3 m, turning and returning to sitting [45]. The TUG test was reported in thirteen reviews (Table 3). Three reviews demonstrated favourable findings [9, 12, 29], four reviews reported unclear or inconsistent findings [24, 26, 27, 35], five reviews demonstrated not favourable findings [10, 13, 20, 38, 42], and one review reported no extractable data on TUG’s ability to predict falls [19]. Across all review studies, the evidence was inconsistent on the ability of the TUG to predict falls. There is some evidence, however, from some subgroup analysis that the TUG may have a role in fall prediction for the lower functioning older adult population [13, 38].

**Table 3:** Summary table of the Timed Up and Go test as a falls assessment tool

Review	Review characteristics	Risk of bias	Summary of key findings	Interpretation
Ambrose [19]	Narrative No details on characteristics	High	No data to extract	Unclear
Lee [29]	Systematic review without meta-analysis (*n* = 4) Mixed settings	High	Community dwelling older people (*n* = 2): - TUG > 12.3 s demonstrated 83.3% sensitivity, 96.6% specificity, 95.9% positive predictive value, 85.8% negative predictive value. - TUG> 20 s 90% sensitivity, 22% specificity, 45% positive predictive value, 75% negative predictive value Acute Inpatient rehabilitation (*n* = 1): - AUC 0.58 (95% CI 0.53–0.63) Outpatient stroke clinics (*n* = 1): - 63% sensitivity, 58% specificity, 58% positive predictive value, AUC = 0.70 (95% CI 0.60–0.81)	Favourable for community-dwelling older adults
Nakamura [20]	Narrative (*n* = not reported) No details on characteristics	High	TUG was reported as one of the most commonly used tests, but do not report predictive ability.	Not favourable
Park [12]	Meta-analysis (*n* = 5, 427 participants) Community-dwelling	High	Pooled sensitivity was 0.76 (95% CI 0.68–0.83), and article heterogeneity was 0.0% (χ2 = 2.20, *P* = .85). Pooled specificity was 0.49 (95% CI 0.43–0.54) and heterogeneity among the articles was high, 94.8% (χ2 = 95.87, *P* < .001). The sROC AUC was 0.80 (SE = 0.04)	Favourable
Schoene [13]	Meta-analysis (*n* = 53) Mixed settings	High	Ddiagnostic accuracy poor to moderate across studies and settings. Pooled estimate of mean difference between fallers and non-fallers in the healthy, higher-functioning samples was 0.63 seconds (95% CI 0.14–1.12, *P* = .01), and the heterogeneity was moderate (v2 = 12.6,(df) = 6,*P* = .05;I2 = 52%) Pooled estimate of mean difference between fallers and non-fallers in studies that included a mix of higher- and lower-functioning people living independently was 2.05 seconds (95% CI 1.47–2.62,*P* < .001), and the heterogeneity was substantial (v2 = 50.7,df = 20,*P <* .001;I2 = 61%) Pooled estimate of the mean difference between fallers and non-fallers in institutional settings was 3.59 seconds (95% CI 2.18–4.99,*P* < .001), and there was no sign of heterogeneity (v2 = 7.7,df = 8,*P* = .47;I2 = 0%)	Not favourable/ favourable for less healthy, lower-functioning groups
Scott [35]	Systematic review without meta-analysis (*n* = 2) Mixed settings	High	Community (*n* = 2): IRR = 0.90 IRR = 0.56 Long term care (*n* = 1): IRR = 0.56	Inconsistent
Beauchet [24]	Systematic review without meta-analysis (*n* = 11) Mixed settings	Unclear	Retrospective studies (*n* = 7): TUG associated with past falls history in all 7 studies Prospective studies (*n* = 4): 3 with no significant association to falls and no significant prediction of falls (2 inpatient, 1 community)) 1 with positive association and prediction of falls in community dwelling	Inconsistent
Dolatabadi [26]	Systematic review without meta-analysis (*n* = 4) Older adults with diagnosis of dementia	Unclear	Successful predictor of future falls (*n* = 2) No predictive value (*n* = 2)	Inconsistent
Lusardi [9]	Meta-analysis (*n* = 12) Community-dwelling	Unclear	TUG > 7.4 s positive likelihood ratio 1.6, negative likelihood ratio 0.7, posttest probability with a positive test 41%, posttest probability with a negative test 23% TUG > 12 s positive likelihood ratio 2.1, negative likelihood ratio 0.8, posttest probability with a positive test 47%, posttest probability with a negative test 25%	Favourable
Barry [10]	Meta-analysis (*n* = 10) Community-dwelling	Low	Logistic regression analysis indicated that the TUG score is not a significant predictor of falls (OR = 1.01, 95% CI 1.00–1.02, *p* = 0.05).	Not favourable
Chantanachai [38]	Meta-analysis (*n* = 16) Older people with cognitive impairment living in the community	Low	Mean difference in meta-analysis fallers vs non fallers TUG 2.20 (−1.42, 5.82), *p* = 0.23 (*n* = 4)	Not favourable
Eagles [27]	Systematic review without meta-analysis (*n* = 1) Emergency department	Low	One study was reported as assessing TUG and falls but no results for falls prediction given. 38% of participants unable to complete TUG.	Unclear
Muir [42]	Meta-analysis (*n* = 1*) Community-dwelling	Low	No data to extract but indicates non-significant findings for falls risk	Not favourable

*Abbreviations: AUC* Area under the curve, *CI* Confidence interval, *df* degrees of freedom, *IRR* Incidence rate ratio, *n* number of included studies, *OR* Odds ratio, *SROC* summary receiver operating characteristic, *TUG* Timed Up and Go. *This study did meta-analyses, but not on TUG, which was only reported in one paper

#### Berg balance scale

The BBS is a balance test with a series of 14 balance tasks that assess a person’s ability to safely balance. Tasks include sitting-to-standing, turning 360 degrees and standing on one leg [46]. The BBS was reported in nine review papers (Table 4).

**Table 4:** Summary table of the Berg Balance Scale test as a falls assessment tool

Review	Review characteristics	Risk of bias	Summary of key findings	Interpretation
Lee [29]	Systematic review without meta-analysis Study (*n* = 4) Mixed settings	High	Community dwelling older people (*n* = 1): - 61% sensitivity, 53% specificity, 49% positive predictive value, AUC = 0.59 Outpatient stroke clinics (*n* = 3): - 69% sensitivity, 65% specificity, 64% positive predictive value, 70% negative predictive value, AUC = 0.69 (0.58–0.80) - 85% sensitivity, 49% specificity, 55% positive predictive value, 83% negative predictive value Cut off < 49, 83% specificity, 91% specificity, 71% positive predictive value, 95% negative predictive value	Favourable for outpatient stroke population
Nakamura [20]	Narrative review No details on characteristics	High	No data to extract	Unclear
Neuls [33]	Systematic review without meta-analysis (*n* = 9) 4 studies with adults with neurological disorders	High	Sensitivity ranges from 25% t0 95.5% Specificity ranged from 20.8 to 100% Calculated cut-off scores ranging from 33 to 54.	Not favourable
Park [12]	Meta-analysis (*n* = 5, 427 participants) Community-dwelling	High	Pooled sensitivity was 0.73 (95% CI 0.65–0.79). Heterogeneity among studies was high (82.7%; χ2 = 23.09, *P* = .0001). Pooled specificity was 0.90 (95% CI 0.86–0.93), and heterogeneity among articles was low (31.9%; χ2 = 5.87, *P* = .21). sROC AUC was 0.97 (standard error [SE] = 0.02)	Favourable
Scott [35]	Systematic review without meta-analysis (*n* = 4) Mixed settings	High	Community (*n* = 3): - reported in one study as 53% sensitivity and 96% specificity Supportive housing (*n* = 1): - significant predictor with score < 45 indicating a relative risk for multiple falls over the next 12 months. Acute: no data to extract	Inconsistent
Dolatabadi [26]	Systematic review without meta-analysis (*n* = 1) Older adults with diagnosis of dementia	Unclear	One study reported on BBS and no significant findings reported.	Not favourable
Lusardi [9]	Meta-analysis (*n* = 4) Community-dwelling	Unclear	BBS < 50 points, positive likelihood ratio 3.4, negative likelihood ratio 0.7, posttest probability with a positive test 59%, posttest probability with a negative test 23%. Sensitivity 41% and specificity 88%	Favourable
Lima [11]	Systematic review without meta-analysis (*n* = 8) Mixed settings	Low	BBS low to moderate sensitivity achieving its best value of 67% for 6-month using a cut-off score of 45 points for any falls, and 69% for 12-month follow-up, using a cut off score of 53 points for multiple falls.	Not Favourable
Muir [42]	Meta-analysis (*n* = 1*) Community-dwelling	Low	One study with non-significant results on fall prediction, meta-analysis not completed for this measure.	Not favourable

*Abbreviations: AUC* Area under the curve, *BBS* Berg Balance Scale, *CI* Confidence interval, *n* number of included studies, *OR* Odds ratio, *SROC* Summary receiver operating characteristic. *This study did meta-analyses, but not on BBS, which was only reported in one paper

Three reviews demonstrated favourable findings [9, 12, 29], one review reported inconsistent findings [29], and four reviews demonstrated not favourable findings [11, 26, 33, 42], on the BBS ability to predict falls. One review did not report any results to extract [20]. Across all the review papers, the evidence for using the BBS to predict falls was inconsistent, and based on the best available evidence [11, 42], the use of the BBS as a balance assessment used in isolation is not recommended to predict falls. There was some evidence from one review that the BBS may have a predictive role in a stroke clinic population [29].

#### Gait speed

Gait speed is the measurement of the time it takes to complete a walk over a given distance in the participant’s preferred or maximum pace [47, 48] and was reported in ten review papers (Table 5). Seven reviews demonstrated positive findings [22, 26, 29, 30, 34, 35, 41]. One reported low sensitivity on the ability of gait speed to predict falls in community dwelling older adults [29], and one reported that a timed walk was not an independent predictor of falls in long term care settings [35]. One review reported that gait speed did not predict falls in cognitive impaired older adults, however a subgroup analysis showed evidence for gait speed predicting falls [38]. One review reported no data to extract [19]. Different distances were used across the studies including 4, 6, 10, and up to 25 m distances. Two reviews investigated usual gait speed [22, 34]. One review reported mainly preferred walking speed [41]. One review reported that of the eight studies that assessed gait speed, six found slow gait speed under standard conditions to predict falls [38].

**Table 5:** Summary table of Gait Speed as a falls assessment tool

Review	Review characteristics	Risk of bias	Summary of key findings	Interpretation
Ambrose [19]	Narrative	High	No data to extract	Unclear
Abellan Van Kan [22]	Systematic review without meta-analysis (*n* = 4, 9477 participants) Community-dwelling older adults	High	All demonstrated gait speed was an independent predictor of falls or falls related fracture. Gait speed reported as at usual pace.	Favourable
Pamoukdjian [34]	Systematic review without meta-analysis (*n* = 9, 6357 participants) Community-dwelling	High	Recommend evaluating gait speed over a distance of 4 m with a threshold of 1 m/s in a single measure as a screening tool for frailty in older patients with cancer (aged 65 years and older); those with gait speed < 1 m/s over a 4-m distance should then be assessed with a CGA.	Favourable
Lee [29]	Systematic review without meta-analysis (*n* = 2) Mixed settings	High	Community dwelling older people (*n* = 1): - 6-m walk test 50% sensitivity, 68% specificity, 37% positive predictive value Outpatient stroke clinics (*n* = 1): - 10-MWT sensitivity 80%, specificity 58%, positive predictive value 64%, negative predictive value 76%, AUC (95%CI) 0.74 (0.64–0.81)	Favourable for stroke patients
Scott [35]	Systematic review without meta-analysis (*n* = 1) Mixed settings	High	Long term care setting: IRR = 0.88 and not reported as an independent predictor for falls.	Not favourable
Dolatabadi [26]	Systematic review without meta-analysis (*n* = 6) Older adults with diagnosis of dementia	Unclear	Gait speeds were often found to differentiate between faller and non-faller in a dementia population. No specific synthesis of data to extract from the review.	Favourable
Chantanachai [38]	Meta-analysis (*n* = 18) Older people with cognitive impairment living in the community	Low	Gait speed −0.07 (−0.28,-0.06) (4 studies, (*p* = 0.46) Of the eight studies that assessed gait speed, six found slow gait speed under standard conditions to predict falls	Not favourable
Ganz [28]	Meta-analysis (*n* = 15) Community-dwelling older adults	Low	Taking more than 13 seconds to walk 10 m predicts recurrent falls with about the same LR as perceived mobility problems (LR, 2.0; 95% CI, 1.5–2.7)	Favourable
Marin-Jimenez [30]	Systematic review without meta-analysis (*n* = 25, 2 systematic reviews) Healthy community-based population older than 18 years. Sub population > 65 years old	Low	Strong evidence for slower gait predicting falls in adults over 60 years (seven studies+ seven studies from systematic reviews) Three studies did not find an association between gait speed test and falls. 6 m walk test reported	Favourable
Menant [41]	Meta-analysis (*n* = 30) Mixed settings	Low	Pooled MD (95% CI) for gait speed between fallers and non-fallers (0.069 (0.045–0.094). Findings indicate single and dual task tests of gait speed are equivalent in the prediction of falls. Slower gait speeds under both single and dual-Task conditions significantly discriminate between fallers and non-fallers. The majority of included studie reported self selected gait speed with two studies reporting unclear specifications.	Favourable

*Abbreviation: AUC* Area under the curve, *CI* Confidence interval, *CGA* Comprehensive geriatric assessment, *IRR* Incidence rate ratio, *n* number of included studies, *MD* Mean difference, *SROC* Summary receiver operating characteristic, *10-MWT* 10 m walking test

Details on the gait speed protocol was lacking in three reviews [19, 26, 35]. The best available evidence suggested that gait speed was a useful measure in predicting falls in community dwelling older adults.

#### Dual task assessments

Dual task assessments are the combination of a physical task (such as walking) and either a second physical task (such as holding an object) or a cognitive task (such as counting) [49] and was reported in seven review papers (Table 6). In detail, four reviews demonstrated favourable findings [24, 32, 41], two review reported unclear findings [23, 37], and one review demonstrated not favourable findings on the ability of dual task testing to predict falls [36]. Evidence for the ability of dual task testing to predict falls over single balance tests was inconsistent; however, the best available evidence suggested that dual task testing had the ability to predict falls. The optimal type of dual task test is still unclear.

**Table 6:** Summary table of Dual Task Assessments as falls assessment tools

Review	Review characteristics	Risk of bias	Summary of key findings	Interpretation
Zijlstra [37]	Systematic review without meta-analysis (*n* = 2) Community-dwelling	High	Two prospective studies suggested that dual balance tasks may have added value for fall prediction over single balance tasks. The low sensitivity (i.e., 55%) reported for fall prediction indicates that only a part of all fallers were identified by the dual-task assessment. Balance tasks included: standing on a force platform, timed up and go, gait speed. Cognitive tasks included: sentence completion, counting backwards verbal response, answering questions.	Inconsistent
Bayot [23]	Systematic review without meta-analysis (*n* = 30) Community-dwelling	Unclear	Promising added value of dual tasks including turns and other transfers, such as in the Timed Up and Go test, for prediction of falls.	Inconsistent
Beauchet [23]	Meta-analysis (*n* = 15) Mixed settings	Unclear	Pooled OR for falling was 1.62 (95% CI 0.96–2.72) for retrospectives studies and 6.84 (95% CI 3.06–15.28) for prospective studies, when subjects had changes in gait or attention-demanding task performance whilst dual tasking. The pooled odds ratio for falling when analysis included all studies was 5.3 (95% CI 3.1–9.1). Walking task incldued: Timed Up and Go and usual gait speed. Attention demanding tasks included: conversations, arithmetic tests carrying a glass of water.	Favourable
Yang [36]	Systematic review without meta-analysis (*n* = 26) Community-dwelling	Unclear	Both static and walking balance assessment tools had good reliability but were not useful to predict falls. In most of the studies, the participants were living independently and had normal cognition. The psychometric properties of dual-task assessment tools may differ depending on the cognitive status. Reviews included primary task of standing or walking balance and secondary task included mental tracking, verbal fluency, working memory, reaction time and discrimination and decision making.	Not favourable
Chantanachai [38]	Meta-analysis (*n* = 16) Older people with cognitive impairment living in the community	Low	Association between poor dual task performance and falls (*n* = 1)	favourable
Muir-Hunter [32]	Systematic review without meta-analysis (*n* = 7) Community-dwelling	Low	Association between dual-task test performance and future fall risk reported. Dual tasks included in the reviews: Primary tasks included gait speed stepping task and postural sway. Secondary tasks included cognitive activities such as verbal fluency tests and motor activity such as carrying a tray with a cup. Changes in gait performance under dual-task testing are associated with future fall risk, and this association is stronger than that for single-task conditions.	Favourable
Menant [41]	Meta-analysis (*n* = 30) Mixed settings	Low	Dual tasks primarily included walking test with secondary cognitive task. Single task and dual task tests across all domains significantly discriminated between fallers and non-fallers (< 0.05). The pooled MD (95%CI) for gait speed between fallers and non-fallers in the single task (0.069 (0.045 0.094) was not significantly different to that in the dual task condition (0.074 (0.046–0.103)	Favourable

*Abbreviations: CI* Confidence interval, *n* number of included studies, *MD* Mean difference, *OR* Odds ratio*This study did meta-analyses, but not on dual task, which was only reported in one paper

#### Single leg stance

The single leg stance test is a single leg standing balance test [50] and was reported in five reviews (Table 7). One review reported favourable findings [39]. Three reviews reported unclear findings on its ability to predict falls [9, 40, 42] and one review demonstrated not favourable findings [31]. Overall, the evidence was inconsistent for the ability of the single leg stance to predict falls.

**Table 7:** Summary table of the Single Leg Stance test as a falls assessment tool

Review	Review characteristics	Risk of bias	Summary of key findings	Interpretation
Chen-Ju Fu [39]	Meta-analysis (*n* = 15) Elderly aged over 65 years who can walk without assistance	High	Maximal standing time identified with high heterogeneity (I2 = 80%) and significant group difference (−6.21 seconds [−10.60–-1.82], *p* = 0.006,) (*n* = 3)	Favorable
Lusardi [9]	Meta-analysis (*n* = 5) Community-dwelling	Unclear	Posttest probability of falling based on SLS time < 6.5 Positive likelihood ratio1.9, negative likelihood ratio 0.9. Posttest probability in positive test 45%, posttest probability if negative test 28%. Sensitivity 19%, specificity 90% Posttest probability of falling based on SLS time < 12.7. Sensitivity 63%, specificity 49%	Inconsistent
Kozinc [40]	Meta-analysis (*n* = 18) Mixed settings	Unclear	Sensitivity moderate to high for single-leg Center of Pressure velocity measures (70–78%), and moderate for single-leg stance time (51–67%). Specificity high only for single-leg stance time in one study (89%) and low to moderate in other studies (43–67%).	Inconsistent
Omana [31]	Meta-analysis (*n* = 21) Community-dwelling	Unclear	The ranges of sensitivity and specificity were 0.51 and 0.61 Sensitivity and specificity for recurrent falls were 0.33 and 0.712, respectively (*n* = 6)	Not favorable
Muir [42]	Meta-analysis (*n* = 5) Community-dwelling	Low	Significant association for increased falls risk found in 1 study, no specific data to extract. No other results for remaining studies reported.	Inconsistent

*Abbreviations: n* number of included studies, *SLS* Single Leg Stance, *SLST* single-leg stance test

#### Functional reach test

The Functional Reach Test is a functional balance test [51] and was reported in nine review papers (Table 8). Six review papers demonstrated favourable findings [9, 20, 26, 31, 35, 39], and three reported not favourable findings on the ability of the Functional Reach Test to predict falls [40, 42, 43]. The evidence across all the reviews was inconsistent for the predictive ability of the Functional Reach Test.

**Table 8:** Summary table of the Functional Reach test as a falls assessment tool

Review	Review characteristics	Risk of bias	Summary of key findings	Interpretation
Chen-Ju Fu [39]	Meta-analysis (*n* = 15) Elderly aged over 65 years who can walk without assistance	High	low heterogeneity (I2 = 0%) and significant group difference (−3.44 cm [−4.60–-2.28], *p* < 0.001, between the two studies.	Favorable
Nakamura [20]	Narrative No study number or characteristics to extract	High	Reported as one of the most common tests and reported as having predictive ability but no results given.	Unclear
Scott [35]	Systematic review without meta-analysis (*n* = 7) Mixed settings	High	Community (*n* = 4): - reported in 1 study as 73% sensitivity and 88% specificity. Long term care (*n* = 2): - no data to extract Acute (*n* = 1): - reported in 1 study as 76% sensitivity and 34% specificity.	Favourable
Dolatabadi [26]	Systematic review without meta-analysis (*n* = 1) Older adults with diagnosis of dementia	Unclear	Significant findings reported in a dementia population (*p* = 0.02)	Favourable
Kozinc [40]	Meta-analysis (*n* = 17) No details of characteristics	Unclear	SMD (95%CI) -0.33 (−0.62, −0.04), *p* = 0.03, positive values indicate a higher value in fallers	Not favourable
Lusardi [9]	Meta-analysis (*n* = 2) Community-dwelling	Unclear	Functional reach distance < 22 cm **points**, positive likelihood ratio 7.9, negative likelihood ratio 0.5, posttest probability with a positive test 77%, posttest probability with a negative test 17%. Sensitivity 55%, specificity 93%.	Favourable
Omana [31]	Meta-analysis (*n* = 21) Community-dwelling	Unclear	For the outcome of any fall, the respective ranges of sensitivity and specificity were 0.73 and 0.88 for the FRT, 0.47 to 0.682 and 0.59 to 0.788 for the modified FRT, (*n* = 8)	Unclear
Rosa [43]	Meta-analysis (*n* = 5) Mixed settings	Unclear	FRT was not capable of predicting falls (*p* = 0.098). The group of older adults who had not fallen presented values 2.30 cm greater (95% CI −0.43-5.04) than those who had fallen in the follow-up period. There is evidence to support the use of the FRT to assess dynamic balance but not to support its use to predict falls.	Not favourable
Muir [42]	Meta-analysis (*n* = 3) Mixed settings	Unclear	No data to extract but indicates non-significant findings.	Not favourable

*Abbreviations: CI* Confidence interval, *FRT* Functional Reach Test, *n* number of included studies included, *SDM* Standardized mean difference

#### Tinetti/ performance-oriented mobility assessment (poma)

The Tinetti test and the POMA test are task-oriented balance tests [52] and were reported in eight review papers (Table 9). Two review papers demonstrated positive findings [19, 38], five review papers reported unclear findings [12, 20, 26, 31, 42], and one review paper reported not favourable findings on the ability of the Tinetti test or POMA to predict falls [9]. There were inconsistent findings across all the reviews on the predictive ability of the Tinetti and POMA test.

**Table 9:** Summary table of the Tinetti or Performance-Oriented Mobility Assessment as falls assessment tools

Review	Review characteristics	Risk of bias assessment	Summary of key findings	Interpretation
Ambrose [19]	Narrative No study number or characteristics to extract	High	No extracted data just described with 2 references: “A reliable and valid clinical test to assess static, dynamic, reactive and anticipatory balance, ambulation and transfers. It has been validated in community-dwelling older people”.	Unclear
Nakamura [20]	Narrative No study number or characteristics to extract	High	Statement in review “The sensitivity of the POAB allows the practitioner to identify that there is a problem, but does not provide enough information on which to base a treatment”	Unclear
Park [12]	Meta-analysis (*n* = 2, 284 participants) Mixed settings	High	The pooled sensitivity was 68% (95% CI 56–79%) and heterogeneity between the articles was 0.0% (χ2 = 0.32, *P* = .57); the pooled specificity was 56% (95% CI 49–62%) and heterogeneity between the articles was high, 79.2% (χ2 = 4.80, *P* = .03)	Inconsistent
Dolatabadi [26]	Systematic review without meta-analysis (*n* = 1) Older adults with diagnosis of dementia	Unclear	POMA was used less frequently in studies with dementia than the instrumented gait, balance measures, and were not as successful in retrospective and prospective studies distinguishing fallers from non-fallers.	Inconsistent
Lusardi [9]	Meta-analysis (*n* = 5) Community-dwelling	Unclear	Scoring less than 25 points (positive test) increased posttest probability to 42%. Scoring more than 25 points (negative test) decreased posttest probability to 23%. Sensitivity53%, specificity 69%	Not favourable
Omana [31]	Meta-analysis (*n* = 21) Community-dwelling	Unclear	For the outcome of any fall, the respective ranges of sensitivity and specificity were 0.076 to 0.615 and 0.695 to 0.97 for the POMA, 0.27 to 0.70 and 0.52 to 0.83 for the modified POMA (*n* = 12)	Inconsistent
Chantanachai [38]	Meta-analysis (*n* = 16) Older people with cognitive impairment living in the community	Low	Association between poor performance in POMA and falls (*n* = 1)	favourable
Muir [42]	Meta-analysis (*n* = 3) Community-dwelling	Low	Significant associations for increased fall risk were found for POMA in 3 studies, data not reported.	Inconsistent

*Abbreviations: CI* Confidence interval, *n* = number of included studies, *POAB* Performance-Oriented Assessment of Balance, *POMA* Performance-Oriented Mobility Assessment, *This study did meta-analyses, but not on POMA, which was only reported in one paper

#### Tandem gait and stance

Tandem gait and stance is a standing balance test and a heel to toe walking test [53] and was reported in eight review papers (Table 10). One review paper demonstrated favourable findings in tandem stand [39]. Two review papers concluded that tandem walk was a significant predictor of falls [28, 42], and one review demonstrated that only tandem walk had the ability to predict falls [9]. Five review papers reported unclear associations [9, 26, 35, 40, 42], and one review reported that the test did not predict falls [27]. The findings across the reviews were inconsistent on the ability of the tandem gait to predict falls. However, tandem walk showed promising results in selecting the population in need of a further evaluation [ref].

**Table 10:** Summary Table of the Tandem Gait and Stance test as a falls assessment tool

Review	Review characteristics	Risk of bias assessment	Summary of key findings	Interpretation
Chen-Ju Fu [39]	Meta-analysis (*n* = 15) Elderly aged over 65 years who can walk without assistance	High	Maximal standing time of the tandem stance test was reported with low heterogeneity (I2 = 0%) and significant group difference (−3.84 seconds [−5.49–-2.18], *p* < 0.001, (*n* = 2)	Favorable
Scott [35]	Systematic review without meta-analysis (*n* = 1)	High	Community dwelling: Sensitivity 55%, specificity 94%	Inconsistent
Dolatabadi [26]	systematic review without meta-analysis	Unclear	No data to extract	Inconsistent
Kozinc [40]	Meta-analysis (*n* = 3) Mixed settings	Unclear	Sensitivity was moderate for single-leg stance time (51–67%). The specificity was high only for single-leg stance time in one study (89%) and low to moderate in other studies (43–67%).	Inconsistent
Lusardi [9]	Meta-analysis (*n* = 3) Community-dwelling	Unclear	Tandem Stance (*n* = 2): Posttest probability of falling on the basis of tandem stance time positive likelihood ratio 1.3, negative likelihood ratio 0.2, post-test probability with a positive test 41%, post-test probability with a negative test 23%, sensitivity 56%, specificity 65% Tandem walk (*n* = 1) Tandem walk (able/unable) positive likelihood ratio 1.6, negative likelihood ratio 0.7, post-test probability with a positive test 36%, post-test probability with a negative test 8%, sensitivity 96%, specificity 23%	Inconsistent/favourable for tandem walk
Eagles [27]	Systematic review without meta-analysis (*n* = 1) Emergency department	Low	Unable to perform tandem gait: 59%. No association between ability to perform tandem gait and self-report falls in 90 days (*p*-value = 0.526)	Not favourable
Ganz [28]	Systematic review without meta-analysis (*n* = 1) Community-dwelling	Low	Inability to perform a tandem walk test (i.e., inability to walk with the heel of one foot touching the toe of the next over 2 m) (LR, 2.4; 95% CI 2.0–2.9) Inability to perform a tandem stand predicts the occurrence of 1 or more falls (LR, 2.0; 95% CI 1.7–2.4)	Favourable
Muir [42]	Meta-analysis (*n* = 13) Community-dwelling	Low	Significant associations for increased fall risk were found for tandem walk for 5 out of the 6 studies. Not data reported. Statistically significant associations for increased falls risk for tandem stand for 4 out of the 9 studies. No data reported.	Favourable for tandem walk. Inconsistent for tandem stand

*Abbreviations: CI* Confidence interval, *LR* Likelihood Ratio, *n* number of included studies

### Chair stand test (cst)

The CST measures the ability to get up from chair without using arms, time taken to get up five times, or number of chair stands over 30 seconds, and was reported in five review papers (Table 11). One review paper demonstrated favourable results [9], 3 papers reported unclear results [19, 35, 38],, and one review reported inconsistent findings on the ability of the CST to predict falls [39]. Overall, the evidence was inconsistent for the ability of CST to predict falls.

**Table 11:** Summary table of the chair stand test as a falls assessment tool

Review	Review characteristics	Risk of bias assessment	Summary of key findings	Interpretation
Ambrose [19]	Narrative No study number or characteristics to extract	High	No extractable data	Unclear
Scott [35]	Systematic review with no meta	high	Sensitivity NS specificity NS IIR 0.63 In one study in long-term care.	Unclear
Chen-Ju Fu [39]	Meta-analysis	High	7805 subjects revealed significant difference in the complete time of the 5-time sit-to-stand test between the two groups (mean difference [faller – non-faller] = 1.90 seconds [95% CI: 0.98–2.82], *p* < 0.001,. However, inconsistent results with high heterogeneity (I2 = 87%) was also detected amongst the included studies, with only one study didn’t favor the non-faller group.	Inconsistent
Lusardi [9]	Meta-analysis (*n* = 3) Community-dwelling	Unclear	For those requiring 12 seconds or more to complete the 5 times sit-to-stand test (5TSTS) (positive test), the PoTP = 41%. For those able to complete this task in less than 12 seconds (negative test), the PoTP = 20%. These findings are derived from data in 1 Level I72 and 2 Level II57,77 prospective studies with a combined sample of 3319 participants.	Favourable
Chantanachai [38]	Meta-analysis	Low	No meta-analysis data	Unclear

*CI* Confidence interval, *LR* Likelihood Ratio, *n* number of included studies, *NS* Not Specified. *IIR* Inter-rater reliability,*This study did meta-analyses, but not timed chair stand, which was only reported in one paper

## Discussion

### Summary

This umbrella review aimed to systematically and critically appraise the evidence on gait, balance, and functional mobility assessments used to predict falls for older adults. A total of 31 review papers were identified, which were mainly systematic reviews without meta-analysis and of low quality with high risk of bias. There were inconsistencies in the findings across all the review papers. The present umbrella review determined that there is not one single gait, balance, and functional mobility assessment that can be used in isolation to predict falls in community-dwelling older adults. The TUG was the most frequently assessed single test for falls prediction, but the findings were inconsistent in its ability to predict falls. There is, however, favourable evidence to suggest that gait speed can be useful in predicting falls and might be included as part of a comprehensive evaluation for older adults. Some positive results were found in dual task assessment as predictors of falls.

### Wider context

Clinical practice guidelines recommend multifactorial interventions to prevent falls in community dwelling older adults who are at an increased risk of falls [6, 14, 54, 55]. Such interventions contain an initial assessment of risk factors for falls and subsequent customised interventions for each patient based on risk factors [56]. The inconsistencies reported across the included review papers highlight the importance of making a clinical judgement including risk factors such as previous falls, cognitive impairment, comorbidity, polypharmacy, activities of daily living, psychological factors, vision impairment, cognitive impairment, and footwear [1, 2].

Clinicians are encouraged to consider individual needs and contexts when evaluating falls risk in older adults. The inconsistencies reported across the review papers of the present umbrella review may have been influenced by the wide range of settings and clinical characteristics included in the individual studies. It is thus challenging to make recommendations for specific settings using the evidence from this review, in light of the degree of heterogeneity across the evidence available. Based on the evidence from this review, we are unable to recommend using the Timed Up and Go, Berg Balance Scale, Chair Stand Test, One Leg Stand, or Functional Reach, alone as single tests for the prediction of falls in older adults. We acknowledge however that these tests have value in assessing mobility and balance limitations and in identifying appropriate targeted interventions.

In post stroke patients, one review reported positive results on the BBS for fall prediction [29], whereas the Functional Reach Test showed positive results in populations with cognitive impairments [26].

Gait speed appeared most promising in fall prediction and has also been associated with other important outcomes like survival and functional capacity [22]. Gait speed is a simple measurement, with no need for expensive equipment and can be performed quickly. The favourable findings in this review indicate gait speed is feasible to complete for community-dwelling older people and older outpatients of stroke clinics. However, gait speed should be assessed through a clearly defined protocol, which specifies the distances to walk or the time allocated to walking, and whether participants walk at their usual speed or maximum speed. One review suggested that the assessment at usual pace gait speed over 4 m might represent a highly reliable instrument to be implemented [22]. Given the number of older people who could benefit from fall risk assessment, an inexpensive assessment tool that can be used in different settings is appealing.

Dual task assessments showed promise in its ability to predict falls, with some evidence suggesting that it was a better predictor of falls than single task assessments [32]. But importantly, differences in testing protocols could have influenced the results, warranting future research with standardised protocols to allow further synthesis of this finding.

The findings from the present umbrella review demonstrate that it is feasible to complete an assessment of gait speed in older adults across a range of settings including the community, long-term care institutions, and rehabilitation settings. Based on the assessment of the falls risk, it is important that interventions are offered to reduce this risk. Exercise programmes have been demonstrated to reduce the rate of falls, particularly for community-dwelling older adults; the most effective programmes include balance and functional mobility exercises [57].

The TUG was the most frequently reported assessment for falls prediction. The TUG is a simple and low-cost test that is easy to administer and has been previously recommended in clinical practice guidelines including the guidelines posited by the American Geriatrics Society/British Geriatrics Society (AGS/BGS) [54, 58]. However, this umbrella review demonstrated that its fall predictive ability was inconsistent. This inconsistency may be explained by heterogeneity in the settings and populations studied, the use of different cut-off times, and the mixed quality of the evidence. One review suggested that the TUG may have a role in predicting falls in lower functioning or institutionalized older adults [13].

### Strengths and limitations

To the authors’ knowledge, this is the first umbrella review examining gait, balance, and functional mobility assessments in the prediction of falls for older adults. Further strengths of this umbrella review included following PRISMA reporting guidelines [15], conducting a comprehensive search for evidence with the support of a research librarian, and clearly stating the objectives beforehand and ensuring transparency with the published protocol. Furthermore, the selection of the included studies was performed in duplicate with a third reviewer resolving any conflicts. The reviewers assessed the quality of the included studies in pairs, using a quality assessment tool designed specifically to assess the risk of bias in systematic reviews, and differences were discussed and resolved between reviewers.

However, this umbrella review has some limitations. The included studies were too heterogeneous to allow for direct comparison of results in a united meta-analysis. In addition, studies involved both prospectively and retrospectively reported falls, which might have contributed to some of the heterogeneity. Also, many of the included review studies were considered to have a high or unclear risk of bias with a lack of clear reporting. This limited the data that could be extracted and synthesised. The differences in the included studies were not statistically assessed, following advice from a statistician that it was not possible to do so, due to heterogeneity between studies. We excluded review papers that were not available in English due to the resources available for the review, therefore, it is possible that the language restriction in the selection of the included studies may have affected the results by introducing a risk of selection bias. We chose to exclude grey literature (e.g., papers that are not published in peer-reviewed journals) to ensure a certain level of quality in the included studies. In this umbrella review no differentiation between falls, multiple fallers or injurious falls were made.

It was not possible from the umbrella review to provide guidance on the critical level of performance in gait speed associated with higher risk of falling. The optimal cut-off in gait speed to predict falls has not been universally defined and accepted, although different cut-offs (eg 1 m/s, 0.8 m/s, 0.6 m/s) have been associated with various adverse health outcomes, including falls. Based on a systematic literature review, an International Academy on Nutrition and Ageing (IANA) expert panel advised to assess GS at usual pace over 4 m and to use the easy-to-remember cut-off point of 0.8 m/s to predict the risk of adverse outcomes [22].

The Short Physical Performance Battery (SPPB) was included in the search terms for this review; however, it was only reported in one review [26], limiting the ability to draw conclusions about its predictive ability in this review. In 2018, the SPPB has been accepted by the European Medical Agency as an assessment tool to assess frailty. In addition, as the SPPB is widely used in geriatric and other medical fields, it is gaining importance. Single studies reported mixed results with regards to fall prediction [59, 60]. Therefore, future research investigating its ability to predict falls and injurious falls is urgently needed.

Further studies are required to investigate the applicability and validity of fall risk assessment tests in different populations with varying functional levels. Different frailty or intrinsic capacity status may influence the fall predictive ability of these tests. Older frailer adults with cognitive or physical impairment may not be able to perform hazardous tasks or follow complex instructions. Low resource settings may lack the equipment and trained staff to perform the more sophisticated tests, despite their potential effectiveness.

We acknowledge that assessing gait, balance, and functional mobility may form only one part of an assessment of falls prediction. Falls prediction approaches may need to further account for the multifactorial nature of falls and the extensive list of factors that can contribute to the risk of falling. The development of multifactorial falls prediction models is an area of ongoing research; however, further work is required before their widespread use is advocated [61].

## Conclusions

Overall, there is not one single gait, balance, and functional mobility assessment alone that can be used in isolation to predict fall risk in community-dwelling older adults. The best available evidence suggests that gait speed is a useful measure in predicting falls and should be considered as part of a comprehensive evaluation of fall risk for older adults. We found that dual task assessments demonstrate some potential to predict falls in older adults, warranting further research in this area.

## Supplementary Information

**Additional file 1.** Search strategy in Medline database.

## Data Availability

The datasets used and/or analysed during the current study are available from the corresponding author on reasonable request.

## Abbreviations

AUC: Area under the curve
BBS: Berg Balance Scale
CI: Confidence Interval
FRT: Functional Reach Test
MD: Mean Difference
n: number of included studies,
OR: Odds ratio
POMA: Performance-Oriented Mobility Assessment
PRISMA: Preferred Reporting Items for Systematic Reviews and Meta-Analyses
PoTP: Posttest probability test
ROBIS: Risk of Bias Assessment Tool for Systematic Reviews
SLS: Single Leg Stance
SROC: Summary receiver operating characteristic,
TUG: Timed up and go test
