# Correction: Predicting falls in older adults: an umbrella review of instruments assessing gait, balance, and functional mobility

**DOI:** 10.1186/s12877-022-03352-5

**Published:** 2022-10-05

**Authors:** D. Beck Jepsen, K. Robinson, G. Ogliari, M. Montero-Odasso, N. Kamkar, J. Ryg, E. Freiberger, T. Masud

**Affiliations:** 1 Geriatric Research Unit, Department of Clinical Research, University of Southern Denmark, Odense, Denmark; 2 Department of Geriatric Medicine, Odense University Hospital, Odense, Denmark; 3 Department of Health Care for Older People (HCOP), Research and Innovation, Queen’s Medical Centre, Nottingham University Hospitals NHS Trust, Nottingham, UK; 4 School of Medicine, University of Nottingham, Nottingham, UK; 5 Gait and Brain Lab, Parkwood Institute, Lawson Health Research Institute, London, ON Canada; 6 Department of Medicine, Division of Geriatric Medicine, Schulich School of Medicine & Dentistry, University of Western Ontario , London, ON Canada; 7 Department of Epidemiology and Biostatistics, Schulich School of Medicine & Dentistry, University of Western Ontario, London, ON Canada; 8 Institute for Biomedicine of Aging, FAU Erlangen-Nürnberg, Nuremberg, Germany

**Correction: BMC Geriatr 22, 615 (2022)**

**https://doi.org/10.1186/s12877-022-03271-5**

After publication of this article [1], the authors reported that the given and family names of the last author were incorrectly structured: ‘Masud Tahir’ should read ‘T. Masud’.

The original article [1] has been corrected.
