# Supplementary material for: Predicting falls in older adults: an umbrella review of instruments assessing gait, balance, and functional mobility
Source: BMC Geriatr. 2022 Jul 25;22:615. doi: 10.1186/s12877-022-03271-5 (PMC9310405; doi:10.1186/s12877-022-03271-5)
Supplement: Supplementary file 1 — Additional file 1. Search strategy in Medline database. [file 12877_2022_3271_MOESM1_ESM.docx]

**Additional File 1**

**Table 1**. Search strategy in Medline database. Search date: November 23^rd^ 2020

| **Search concept** | **#** | **Search strategy** | **Number of results** |
| --- | --- | --- | --- |
| **Older adults** | 1 | Elderly.mp. or Aged/ | 3169992 |
|  | 2 | (Older adj2 (people* or person*)).mp. | 42996 |
|  | 3 | Senior*.mp. | 41795 |
|  | 4 | 1 or 2 or 3 | 3210384 |
| **Gait Assessment** | 5 | Gait Analysis/ or Gait/ | 28210 |
|  | 6 | Postural Balance/ | 23422 |
|  | 7 | (Assessment adj2 (Gait* or Balanc*)).mp. | 2148 |
|  | 8 | (Timed Up and Go Test).mp. | 2560 |
|  | 9 | "Timed up and go test".mp. | 2550 |
|  | 10 | Walking Speed.mp. or Walking Speed/ | 7388 |
|  | 11 | Gait Speed.mp. or Walking Speed/ | 5709 |
|  | 12 | Tandem.mp. | 136915 |
|  | 13 | Berg Balance.mp. | 2220 |
|  | 14 | Short Physical Performance Battery.mp. | 1360 |
|  | 15 | SPPB.mp. | 868 |
|  | 16 | Dynamic Gait Index.mp. | 320 |
|  | 17 | Sit to stand test.mp. | 740 |
|  | 18 | Chair stand test.mp. | 515 |
|  | 19 | leg stand test.mp. | 33 |
|  | 20 | sway.mp. | 6197 |
|  | 21 | Tinetti score.mp. | 37 |
|  | 22 | 5 or 6 or 7 or 8 or 9 or 10 or 11 or 12 or 13 or 14 or 15 or 16 or 17 or 18 or 19 or 20 or 21 | 198276 |
| **Fall Prediction** | 23 | Fall* Predict*.mp. | 272 |
|  | 24 | Predict* of Fall*.mp. | 1369 |
|  | 25 | Fracture* Predict*.mp. | 567 |
|  | 26 | Predict* of Fracture*.mp. | 1849 |
|  | 27 | Accidental Falls/ or Accidental Fall*.mp. | 25022 |
|  | 28 | 23 or 24 or 25 or 26 or 27 | 27728 |
| **Types of Studies** | 29 | Prospective Studies/ or Prospective Stud*.mp. | 622409 |
|  | 30 | Longitudinal Studies/ or Longitudinal Stud*.mp. | 175447 |
|  | 31 | Cohort Studies/ or Cohort Stud*.mp. | 405523 |
|  | 32 | 29 or 30 or 31 | 1081156 |
| **Umbrella Review** | 33 | "Review"/ or Review.mp. | 3367866 |
|  | 34 | Review of Reviews.mp. | 570 |
|  | 35 | Umbrella Review.mp. | 450 |
|  | 36 | Analytic Review.mp. | 1335 |
|  | 37 | 33 or 34 or 35 or 36 | 3367866 |
| **Total** | **38** | **4 and 22 and 28 and (32 or 37)** | **1134** |
